# Oligo-Metastatic Disease in Oncology: Exploring the Limits and the Potential of Genetic Assessment

**DOI:** 10.3390/genes14122131

**Published:** 2023-11-26

**Authors:** Mariachiara Santorsola, Maurizio Capuozzo, Giovanni Savarese, Monica Ianniello, Nadia Petrillo, Marika Casillo, Francesco Sabbatino, Francesco Perri, Francesco Ferrara, Andrea Zovi, Massimiliano Berretta, Vincenza Granata, Guglielmo Nasti, Alessandro Ottaiano

**Affiliations:** 1 Istituto Nazionale Tumori di Napoli, IRCCS “G. Pascale”, Via Mariano Semmola, 80131 Napoli, Italy; mariachiara.santorsola@istitutotumori.na.it (M.S.); f.perri@istitutotumori.na.it (F.P.); v.granata@istitutotumori.na.it (V.G.); g.nasti@istitutotumori.na.it (G.N.); 2 Coordinamento Farmaceutico, ASL-Naples-3, 80056 Ercolano, Italy; m.capuozzo@aslnapoli3sud.it; 3 AMES, Centro Polidiagnostico Strumentale srl, Via Padre Carmine Fico 24, 80013 Casalnuovo Di Napoli, Italy; giovanni.savarese@centroames.it (G.S.); monica.ianniello@centroames.it (M.I.); nadia.petrillo@centroames.it (N.P.); marika.casillo@centroames.it (M.C.); 4 Department of Medicine, Surgery and Dentistry, University of Salerno, 84081 Baronissi, Italy; fsabbatino@unisa.it; 5 Hospital Pharmacist Manager, Pharmaceutical Department, Asl Napoli 3 Sud, Via Dell’amicizia 22, 80035 Nola, Italy; f.ferrara@aslnapoli3sud.it; 6 Hospital Pharmacist, Ministry of Health, Viale Giorgio Ribotta 5, 00144 Rome, Italy; zovi.andrea@gmail.com; 7 Department of Clinical and Experimental Medicine, University of Messina, 98122 Messina, Italy; mberretta@unime.it

**Keywords:** oligo-metastatic disease, NGS, genetics, liquid biopsy

## Abstract

Oligo-metastatic disease (OMD) in the field of oncology denotes a distinct subset of metastatic tumors characterized by less aggressive biological behavior and extended survival times in comparison to their widely metastatic counterparts. While there is a general consensus regarding the existence of OMD, there remains a lack of widely accepted criteria for its a priori identification at the time of presentation. This review delves into the concept of OMD, placing a particular emphasis on the significance of understanding the limitations and potential of genetic assessments. It explores how these aspects are crucial in advancing our comprehension of this phenomenon. In a rapidly advancing era of precision medicine, understanding the intricacies of OMD opens up exciting possibilities for tailored treatment approaches. By elucidating the genetic underpinnings and dynamic nature of this condition, we stand to improve patient outcomes and potentially shift the paradigm of metastatic cancer management.

## 1. Introduction: Epidemiology and Definitions of Oligo-Metastases

Metastasis, the spread of cancer cells from the primary tumor site to distant organs, represents a pivotal hallmark of advanced cancer. In contrast to widespread metastatic disease, oligo-metastases constitute a unique clinical entity characterized by a limited number of metastatic lesions, typically fewer than five, and a notably less aggressive disease course [1]. Patients with oligo-metastatic disease (OMD) often exhibit prolonged survival, offering a distinct therapeutic window for intervention [2]. The concept of oligo-metastases was first introduced by Dr. Samuel Hellman and Dr. Ralph R. Weichselbaum in 1995 [3], challenging the traditional view that metastasis invariably indicates systemic disease and carries a dire prognosis. Its identification is essential, as patients with true OMD may benefit from aggressive local treatments aimed at eradicating all metastatic lesions when feasible, potentially achieving long-term disease control or even cure [1,2].

The epidemiology of OMD varies across different cancer types. It is more commonly observed in certain malignancies, such as colorectal, breast, lung, and prostate cancer, while less frequent in others [1]. The incidence of oligo-metastases is influenced by the tumor’s intrinsic biological characteristics, the patient’s immune response, and the microenvironment of both the primary tumor and metastatic sites. Interestingly, while it was once hypothesized that certain cancers, such as pancreatic adenocarcinoma and small-cell lung cancer, never exhibited an oligo-metastatic behavior, current evidence indicates that OMD can be documented in all cancer types [4,5,6,7,8]. Notably, the incidence of “induced” OMD is rising across all cancers due to advancements in systemic treatments, including biologic drugs, immunotherapies, and integrated therapeutic approaches, which improve outcomes for primary poly-metastatic cancers.

Previous research has adopted a pragmatic and quantitative approach to define OMD. For instance, OMD has been defined as 1–3 metastatic tumors per organ with a size limitation of less than 7 cm [9]. In a more stringent definition, a recent study specified a maximum of 1–5 tumors with a size limit of 5 cm [10]. Nevertheless, attempts to incorporate the “rate of metastatic growth,” which is typically slower in OMD [11], into a quantitative criterion remain challenging and less practical for clinical application. Alternatively, some authors propose defining OMD as the presence of metastatic cancer that allows for curative or radical local interventions (such as surgery or radiotherapy) on all metastatic lesions, regardless of their number or volume [3,12].

A recent consensus study by ASTRO/ESTRO (American Society for Radiation Oncology/European Society for Radiotherapy and Oncology) [13] has provided explicit and practical definitions for various clinical scenarios of OMD. Familiarity with these definitions is essential, not only for standardizing scientific terminology but also because they will necessitate prospective validation and further scientific exploration in the near future. In understanding the classifications and nomenclature of OMD, we discern several distinct categories that shed light on its nuanced nature. Firstly, we encounter “Genuine OMD”, also known as “de novo” OMD. This form represents the purest manifestation of OMD, characterized by a lack of any previous history of poly-metastatic disease. Further differentiation is made between synchronous and metachronous OMD, depending on whether the diagnosis occurs within or after six months of the primary cancer diagnosis, respectively. Some patients present with ‘sync-oligometastatic’ cases [14], in which OMD manifests synchronously with an active primary tumor. These patients have a worse prognosis compared to ‘oligo-recurrence’ cases (see below for the definition of oligo-recurrence). Another category is “induced OMD”, which unfolds as a poly-metastatic cancer transforms into a more constrained state, limited to a small number of metastatic sites (OMD). This transformation typically occurs following systemic treatment and exemplifies the dynamic nature of OMD. Moreover, we observe “repeat OMD”, which comes into play when OMD resurfaces after a prior diagnosis and treatment for OMD. This recurrence underscores the challenges of managing OMD over time. Intriguingly, “repeat” and “induced” OMD may be coupled with diverse imaging dynamics. “Repeat oligo-recurrence” and “induced oligo-recurrence” signify the emergence of new oligo-metastatic lesions either from existing OMD or as a consequence of poly-metastatic disease, respectively. Within this framework, “Oligorecurrence” describes OMD that reappears after initial treatment during a treatment-free interval. “Oligoprogression” denotes the progression of OMD while actively undergoing systemic treatment, adding a layer of complexity to therapeutic decisions. Lastly, “Oligopersistence” highlights the resilience of OMD, persisting even after initial treatment. These classifications not only aid in characterizing OMD but also emphasize the intricate nature of its evolution and treatment considerations.

Despite the recognized clinical significance of oligo-metastases, the lack of universally accepted molecular criteria for their upfront identification poses a substantial challenge. Current diagnostic methods often rely on conventional imaging techniques, such as computed tomography (CT), magnetic resonance (MR), and positron emission tomography (PET), to assess the extent of metastatic spread without delving into molecular specifics.

## 2. Clinical Surrogates for Identification of Oligo-Metastatic Disease: Pragmatic Considerations

OMD is distinguished by its less aggressive biological behavior compared to widely metastatic cancers. This unique characteristic is multifaceted and contributes to the extended survival times observed in patients with oligo-metastases. One key aspect of the less aggressive biological behavior of OMD is the limited number of metastatic lesions. Typically, oligo-metastatic patients present with fewer than five distinct metastatic sites. This limited metastatic burden contrasts sharply with a widely metastatic disease, where multiple lesions infiltrate organs and tissues throughout the body. The restricted spread of cancer cells in oligo-metastatic cases suggests a certain degree of control over the disease’s dissemination. Another hallmark of the less aggressive biological behavior of OMD is the relatively indolent growth of metastatic lesions. These lesions often progress more slowly than their widely metastatic counterparts, allowing for a more protracted clinical course. The reasons for this slower progression are unknown; they could be related to the selective pressures imposed by the immune system and the microenvironment of the metastatic sites [15,16]. These factors may limit the growth and invasiveness of cancer cells, contributing to the less aggressive phenotype. Furthermore, oligo-metastatic lesions frequently exhibit a lower degree of genetic heterogeneity compared to widely metastatic tumors [17,18]. Genetic heterogeneity refers to the presence of diverse genetic alterations within a tumor, which can drive treatment resistance and disease progression. Oligo-metastatic lesions may possess a more homogeneous genetic profile, making them potentially more susceptible to targeted therapies. This reduced genetic complexity aligns with the concept of ‘clonal evolution’ and suggests that oligo-metastatic lesions may have originated from a common ancestral clone within the primary tumor, which possesses lower metastatic potential.

The less aggressive biological behavior of OMD translates into a distinct clinical outcome: prolonged survival. Patients diagnosed with true OMD often experience extended periods of disease-free or progression-free survival, even in the absence of aggressive systemic therapies. This favorable prognosis has spurred interest in identifying and treating oligo-metastatic patients with curative intent. The extended survival times observed in oligo-metastatic patients provide a unique therapeutic window for interventions aimed at achieving local disease control or even complete remission [19,20,21,22]. Unlike widely metastatic disease, where systemic treatments are the primary focus, oligo-metastatic patients may benefit from aggressive local therapies, such as surgical resection or ablative techniques. These interventions target a limited number of metastatic lesions and can lead to long-term disease-free intervals.

While the less aggressive biological behavior and extended survival times associated with OMD are compelling, identifying this clinical entity remains a challenge. The lack of widely accepted criteria for upfront identification poses a significant hurdle in clinical practice. Current diagnostic methods, primarily relying on imaging techniques, may not reliably distinguish between oligo-metastatic and widely metastatic diseases. Moreover, the heterogeneity of cancer types and individual patient characteristics further complicates the identification of oligo-metastatic cases. OMD is more commonly observed in specific cancer types, but it can occur in a broader range of malignancies. The dynamic nature of cancer progression adds to the complexity, as some patients in clinical practice may transition from an oligo-metastatic state to a widely metastatic one over time. Thus, still to date, identifying OMD is necessarily carried out retrospectively, as many patients who undergo radical treatment of oligo-metastases develop aggressive, poly-metastatic diseases within a year, while others never experience disease progression (true OMD).

In the following sections of this manuscript, we will explore the role of genetics in the characterization of OMD. Understanding the molecular underpinnings of oligo-metastases may offer a more reliable and precise method for identification, guiding treatment decisions, and improving patient outcomes. Additionally, we will discuss the limitations of current technical approaches to genetic assessments and explore the potential of dynamic molecular profiling in cancer management.

## 3. Genetic Assessment of Oligo-Metastatic Disease

Genetics plays a pivotal role in comprehending the nature and behavior of metastatic disease in cancer. As mentioned earlier, the less aggressive biological behavior and extended survival times associated with oligo-metastatic tumors suggest the presence of distinct genetic characteristics that differentiate them from widely metastatic cancers.

One of the fundamental concepts in this study of OMD is the importance of genetic profiling. Next-generation sequencing (NGS) has emerged as a powerful tool in cancer genetics, enabling the comprehensive analysis of a tumor’s DNA genomic alterations. NGS allows for the simultaneous examination of multiple genes and genetic pathways, providing a wealth of information about the molecular characteristics of cancer [23,24]. In the context of OMD, NGS offers several advantages. First, it enables the identification of specific genetic alterations that may drive the less aggressive behavior of oligo-metastatic lesions. These alterations may include mutations in genes associated with cell proliferation, angiogenesis, or immune evasion. NGS also facilitates the monitoring of genetic dynamics. However, it is imperative to discuss that despite diligent efforts invested in the realm of genetic sequencing techniques and genotype-phenotype associations meticulously curated to select patients afflicted with oligo-metastatic conditions, there has been an absence of substantial disparities detected between the primary tumors destined to evolve into an oligo-metastatic ailment versus their poly-metastatic counterparts. Several factors may elucidate this phenomenon. Traditional “static” genetic assessments, which involve the characterization of the primary tumor’s genetic makeup, often fail to capture the dynamic changes that occur during cancer progression and metastasis [25,26]. In fact, the solitary and static evaluation of the genetic makeup of the primary tumor may be methodologically flawed, as the pivotal factors governing OMD progression may exhibit a highly dynamic nature. Specifically, the host’s immune system might play a fundamental role in sculpting the neoplastic population, directing it along an oligo-metastatic trajectory [15,16]. In this context, dynamic and comparative assessments (primary tumor vs. metastasis) may offer more insightful information. An indirect indication that this might be a methodologically superior approach arises from the phenomenon of “regression” in *KRAS* mutations observed in select oligo-metastatic patients [27]. This phenomenon would have remained entirely overlooked in the absence of dynamic evaluations of tumor genetics. Studies are needed to unravel the origins of these alterations; are they related to immunological factors, stochastic events, or microenvironmental influences?

In other words, a single genetic snapshot of the primary tumor may not adequately represent the genetic diversity and evolution of metastatic lesions. The most intuitive dynamic assessment involves the analysis of genetic alterations in both the primary tumor and metastatic lesions over time, at least in patients who have undergone surgical removal of all metastatic lesions. Of course, this is an infrequent scenario in clinical practice but not entirely impossible for oligo-metastatic patients. Another plausible explanation for the challenges in delineating the genetic underpinnings of OMD is that the genetic foundations of OMD could potentially reside in genetic events previously considered “low-priority” based on existing knowledge, including genetic polymorphisms or mutations that have not yet been discovered [28,29,30]. It remains conceivable that some of these unrecognized events may already be embedded within the vast troves of big data generated by NGS platforms. In such an eventuality, we would indeed be analogous to the proverbial fool who fixates on the finger while the wise discern the moon. Naturally, in the latter scenario, it becomes imperative to augment both the quantity of specimens under scrutiny and the sophistication of our analytical methodologies. A multidisciplinary collaborative approach with molecular biologists and biostatisticians would be instrumental in achieving this aim.

**Figure 1:**
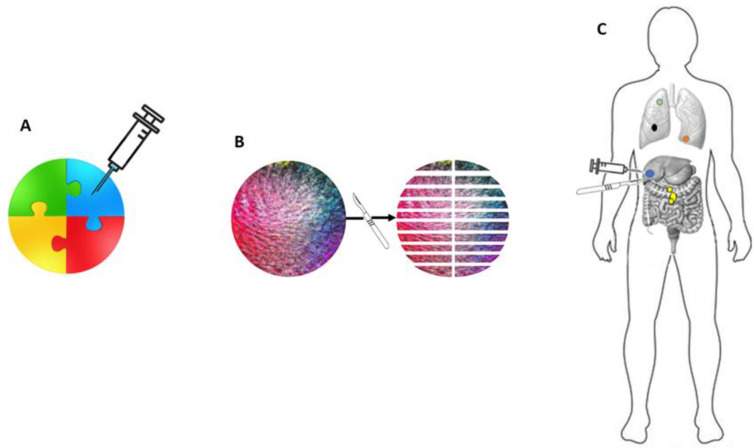
There are technical limitations to genetic assessment in neoplasms. In (**A**), a biopsy of a composite tumor mass is depicted (each color represents different genetic configurations). The biopsy samples only the blue tissue, which represents a limited and non-representative portion of the tumor’s genetic events. The yellow portion, indicative of genetic events associated with aggressive biological behavior, is entirely overlooked. In (**B**), even under an ideal and theoretical condition of complete excision and total sampling of a tumor mass, the landscape of genetic events can still be limited. For instance, the yellow cellular clone present at the upper pole of the mass is found in only one out of 20 of the macroscopic fragments obtained. In (**C**), it is illustrated how a relevant genetic event (in yellow) may occur in an anatomical location distant from partial biopsies or excisions of the tumor mass and could, therefore, be entirely disregarded.

The challenging identification of genetic events driving the oligo-metastatic status might be rooted in the current methodological constraints associated with tissue sampling (Figure 1).

Tumor heterogeneity, both within the primary tumor and among metastatic lesions, presents a significant challenge when it comes to collecting genetic assessments. OMD, similar to other types of cancer, can display intratumoral and interlesional heterogeneity. Intratumoral heterogeneity refers to genetic variations within a single tumor, where distinct subclones of cancer cells may carry unique genetic alterations. Interlesional heterogeneity pertains to differences in genetic profiles among various metastatic lesions within the same patient. Obtaining a biopsy from just one metastatic site may not comprehensively represent the genetic landscape of all metastases. Consequently, relying solely on a single biopsy may result in incomplete or misleading information regarding the genetic characteristics of the disease, potentially complicating the interpretation of genetic data. To address this challenge, multiple biopsies from different metastatic sites may be necessary to capture the full spectrum of genetic alterations. By repeatedly sequencing tumor biopsies from both the primary site and metastatic lesions, researchers can ideally track the evolution of genetic alterations as the disease progresses. This dynamic approach provides valuable insights into the clonal evolution of cancer, revealing the emergence of subpopulations of cancer cells with unique genetic profiles [31,32,33,34]. However, it is worth emphasizing that while tumor biopsies remain a valuable source of genetic information, they are invasive and may not always be feasible, especially when multiple metastatic sites are involved. Furthermore, in some cases, tumors may be located in anatomically challenging areas, making biopsies risky or impractical. Additionally, biopsies are invasive procedures that carry potential complications, including bleeding, infection, and injury to nearby structures. Most importantly, obtaining serial biopsies to monitor genetic dynamics can be logistically challenging and burdensome for patients.

These technical limitations may necessitate the exploration of alternative approaches, such as liquid biopsies, to overcome some of the challenges associated with tissue biopsies. In contrast, liquid biopsies have gained prominence as a less invasive and more accessible means of studying genetic dynamics in cancer. Liquid biopsies involve the analysis of circulating tumor DNA (ctDNA) shed by cancer cells in the bloodstream [35,36,37]. However, the technique allows a comprehensive genetic assessment of both primary and metastatic lesions, or only metastatic lesions if the primary tumor has been removed. While liquid biopsies are particularly useful in depicting the genetic landscape of a cancer or monitoring the emergence of drug-resistant mutations (allowing for timely adjustments in therapeutic strategies), they do not distinguish the genetic trajectories of individual lesions, and some events might go unnoticed simply because they are not released by cancer cells and, therefore, not detectable in peripheral blood. In essence, both solid and liquid biopsies could perpetually represent a genetically non-representative fraction of the heterogeneous neoplastic population. The examination of ctDNA warrants attention as liquid biopsy gains prominence for tumor characterization. While assessing ctDNA is valuable for understanding tumor genetics, it is not without inherent limitations. Principal among these is tumor heterogeneity, where distinct genetic alterations exist across different tumor regions. In fact, intratumoral heterogeneity, both spatial and temporal, introduces additional variations in the genetic composition across different tumor regions and over time [18,33]. Subclonal populations, even with crucial genetic mutations, may not shed detectable levels of DNA into circulation, limiting the comprehensiveness of ctDNA analysis. Technical constraints, including sensitivity thresholds and allele dropout in ctDNA detection methods, can impact result accuracy. Low-frequency mutations or those in subclonal populations may fall below detection limits, portraying an incomplete genomic profile. Moreover, the dynamic nature of tumors, influenced by metabolic changes, selective pressures, and treatments, can alter clonal architecture, further challenging ctDNA representativeness [38]. Finally, apoptosis and necrosis, compared to active release mechanisms, majorly contribute to ctDNA release [39]. Consequently, ctDNA predominantly reflects genetic information from apoptotic or necrotic tumor cells, potentially underrepresenting genomic alterations in quiescent or actively proliferating regions. Addressing these challenges is crucial for enhancing the reliability of ctDNA analysis in characterizing tumors.

Among the latest challenges to be discussed, the collection and analysis of genetic data undoubtedly raise ethical and privacy concerns [40]. Patients may have reservations about genetic testing, particularly if it involves the sharing of sensitive information. Ensuring the secure storage and responsible use of genetic data are essential to maintaining patient trust and confidentiality. Additionally, there are ethical considerations related to the unexpected potential implications of genetic findings. Genetic data may reveal information about a patient’s predisposition to other diseases. Balancing the benefits of genetic profiling with the potential psychosocial and ethical implications requires careful consideration.

Finally, it has been widely acknowledged that investigating the intricate relationship between genotype and phenotype in cancer poses significant challenges. Most cancers typically develop as complex, multi-gene acquired diseases, with a few exceptions like retinoblastoma and Wilms’ tumor, which are inherited forms of tumors. The primary challenge in establishing genotype/phenotype correlations lies in selecting appropriate human cancer models. This is due to the involvement of numerous genes in common conditions such as hypertension, diabetes, allergies, and chronic inflammation, which contribute to the diversity within cancer [41,42,43,44,45]. These concurrent diseases can influence cancer genetics, as certain genes implicated in cancer-related processes, such as proliferation and angiogenesis, are altered in conditions like hypertension and atherosclerotic plaque or are induced by factors like hypoxia, oxidative stress, and inflammation.

In conclusion, while genetic profiling and dynamic assessments offer valuable insights into OMD, several limitations and challenges must be addressed. Tumor heterogeneity, spatial and temporal variability, technical challenges in biopsy collection, interpretation of genetic data, and ethical concerns all impact the utility and implementation of genetic assessments. Overcoming these challenges will require ongoing research, technological advancements, and a multidisciplinary approach involving oncologists, geneticists, and ethicists. Despite these limitations, genetic assessments remain a critical tool in advancing our understanding of OMD.

## 4. Genetics of Oligo-Metastatic Disease

Taking into account the aforementioned limitations, the investigation and identification of the molecular characteristics of OMD constitute a pivotal objective. In the context of metastatic diseases, clinical staging systems are inherently flawed and excessively simplistic. Regrettably, only a limited number of studies have thus far been conducted to explore the specific genetic and biological attributes of OMD.

It was reported that distinct microRNA profiles in OMD patients drive an oligo-metastatic behavior both in vitro and in vivo [46]. Intriguingly, these oligomiRNAs target genes involved in adhesion, invasion, and migration. Even though this research encompassed tumor cells from diverse histologies (colon, small cell lung cancer, non-small cell lung cancer, renal, sarcoma, and ovarian cancer), the majority of oligomiRNAs were mapped to a common locus (14q32), suggesting that shared epigenetic/genetic phenomena underlie OMD. In a comprehensive study on clear cell renal carcinoma, somatic copy number alterations, genetic intra-tumor heterogeneity, and chromosome 9p status were associated with the oligo-metastatic and favorable prognosis of clear cell renal carcinoma. Furthermore, in contrast to the prevailing belief that gene mutations invariably drive cellular transformation and metastasis, certain driver gene mutations (*PBRM1* and *SETD2*) were linked to attenuated progression and OMD [47]. More studies have emerged in colorectal cancer (CRC), where molecular subtyping (“canonical” and “immune” subtypes) [48], regression of key-driver gene mutations (*KRAS*, *PIK3CA*) [16], high levels of T-cell infiltration into metastases, and specific gene mutations (*ERBB2*) have been correlated with the OMD phenotype [15]. In particular, our prior reports [49,50] furnished an evolutionary and dynamic analytical perspective, underscoring that comparing primary and metastatic lesions could aid in identifying genuine de novo oligo-metastatic behavior. Specifically, our findings revealed that patients exhibiting a “regressive” genetic trajectory from primary to metastatic lesions, coupled with high granzyme-B and CD8+ T cell infiltration into the tumor core of metastatic lesions, did not experience relapse within 3 years of follow-up. Conversely, patients lacking these characteristics developed poly-metastatic disease within 1 year after radical resection of all visible lesions. To discern the most prominent and interrelated genes in these patients, we harnessed the Phenolyzer tool [49]. Interestingly, aside from *APC* and *TP53*, *EP300* emerged as one of the top three dominant genes. *EP300* encodes a histone acetyl-transferase responsible for regulating chromatin activity, with the potential to influence crucial cellular processes like proliferation and differentiation [50]. Despite *EP300* mutations being observed in various cancers, including CRC, their role in tumorigenesis remains a topic of debate and contradiction. In addition to genes implicated in proliferation, apoptosis, differentiation, and neoangiogenesis, a significant number of other genes were identified in the subgroup exhibiting “true” de novo OMD. These genes are associated with DNA repair mechanisms, encompassing *MSH3*, *BRCA1*, *ATM*, *POLE*, *BRCA2*, *CHEK1*, and *GLI1*. The heightened microsatellite instability (MSI) and tumor mutational burden (TMB), in conjunction with these genes, may contribute to the heightened immunogenicity of metastases in this patient cohort, who did not develop poly-metastatic disease during subsequent follow-up. Conversely, patients who did progress to poly-metastatic disease after radical resection of all lesions displayed marked mutational divergence, with only one shared gene: *RP11*-*145E5.5*. This gene encodes S-methyl-5′-thioadenosine phosphorylase (MTAP), a player in polyamine biosynthesis [51]. Although the loss of MTAP activity has been hypothesized to play a role in malignant melanoma, its role in CRC, where it appears overexpressed compared to normal mucosa and positively correlated with CRC cell aggressiveness, remains largely unknown [52]. Frequent alterations of genes associated with liver metastasis homing were also observed, including *HSP90AA1*, *NR4A2*, *KDR*, *FLT3*, and *RPS6KB2*. These genes can directly promote cell migration, stimulate epithelial-mesenchymal transition (EMT), and enhance proliferation. Alternatively, they can exert pleiotropic effects, including protein stabilization, epigenetic modifications, and protein synthesis. In patients with lung-limited oligo-metastasis, genetic changes were identified in *EpCAM* (Epithelial cell adhesion molecule), *TP53*, *caspase-8*, and *ERBB2*. These alterations are considered significant and are frequently shared among patients. Notably, both EpCAM and caspase-8 play a role in regulating cell proliferation, migration, and adhesion to lung tissue [53,54]. Hence, their alteration could, at least in part, contribute to the lung homing of metastatic cancer cells. *ERBB2* was also frequently mutated, exhibiting a non-synonymous coding variant, p.P1170A, which may impact the spatial conformation of the tail region and affect tyrosine kinase activity [55]. *ERBB2* amplification has been extensively studied in cancer, but little is known about the role of point mutations. Intriguingly, in ERBB2-overexpressing breast cancer cell lines, lung colonization is predominant and mediated by SPARC (a secreted protein acidic and rich in cysteine) [56,57]. Furthermore, our previous research has indicated that certain genes associated with type 2 diabetes (T2D) may also play a role in the malignant phenotype of OMD in CRC [58]. Specifically, certain variants linked to T2D, such as *HNF1A* p.I27L, *IDE3* p.T105A, *IRS1* p.S892G, and *INSR* p.A2G, though considered benign, could influence the activity of related proteins. These effects may also manifest through changes in 5′-UTR or intron variants that impact transcription activity or alternative splicing. The genetic results of OMD in CRC have shown that these genetic variants (polymorphisms) are less prevalent compared to poly-metastatic disease. This observation introduces added complexity to the cancer transformation processes and the phenotype associated with OMD. Importantly, the effects of these variations remain largely unknown and are underappreciated from both a functional and clinical standpoint. Notably, diabetes-related *TCF7L2* variations were not detected in the observed group of OMD patients with CRC. TCF7L2 is a transcription factor that plays a pivotal role in various pathways linked to CRC and serves as a key player in the Wnt pathway [59]. The *TCF7L2* gene is strongly linked to T2D and resides on chromosome 10q25.3, with rs7903146 representing one of the most prevalent single nucleotide polymorphisms within the *TCF7L2* gene. Finally, we previously observed a higher prevalence of the HLA-C7 allele in OMD from CRC patients. This data could support the enhanced presentation of specific tumor antigens and align with specific immunological phenomena underlying the oligo-metastatic status [60].

The genetics of cancer can be shaped by the immune system, and the hypothesis that OMD is dependent on immune factors is not only intriguing but also marked by significant biological plausibility. In other words, host-related immunological factors, such as a more efficient capacity for tumor antigen presentation (adaptive immunity) and/or enhanced efficiency in natural immunity-mediated elimination, could contribute to rendering the tumor progression less virulent. Concerning the former aspect, although specific T lymphocytes for the antigen typical of OMD have not been identified, there are indirect data. In a study on breast cancer, despite not comparing with poly-metastatic disease, high CD3+ lymphocyte infiltration assessed by immunohistochemistry (IHC) (>10%, percentages of positive cells per slide) in intratumoral oligo-metastatic lesions correlated with longer progression-free survival and overall survival [61]. Furthermore, in liver oligometastasis from colorectal cancer, a high intratumoral CD8/CD3 ratio (>0.32; significant stained cells were quantified by identifying and calculating subtype-specific cell numbers per field based on positivity and total cell count through four-color IHC) correlated with significantly longer 3-year relapse-free survival and 3-year overall survival rates than those with low intratumoral CD8/CD3 ratios [62]. Similarly, in oligo-metastatic lesions evolving towards a poly-metastatic disease after surgical removal of single-nodule liver metastases from CRC, patients exhibited scarce granzyme-B CD3+ CD8+ cells (27.6 + 2.7 per mm^2^) compared to those without relapse at three years (58.6 + 8.6 per mm^2^; *p* = 0.0270) assessed through IHC [16]. Moreover, oligo-metastatic patients demonstrated peripheral CD3+/CD8+ lymphocytes capable of efficiently recognizing and eliminating differentially *KRAS*-mutated CRC cells. The cytotoxic properties of these patients seemed consistent with the mutational and clinical course of the neoplastic progeny, comparing the genetic and tumor microenvironment characteristics of primary and matched metastases [60]. Obviously, the interaction between these cytotoxic T lymphocytes is necessarily based on major histocompatibility complex (MHC)-restricted tumor reactivity, eventually capable of controlling the tumors. As previously anticipated, another anti-tumor immune response involves the activation of nonspecific, MHC-independent lysis of tumor cells by natural killer (NK) cells. Literature data for OMD are limited, primarily confined to highly specific clinical settings, such as the treatment of oligometastases with stereotactic body radiotherapy (SBRT). In some instances, an elevated count of peripheral activated NK cells, assessed through multispectral flow cytometry, is observed immediately after the initial SBRT dose in breast cancer [63]. However, a recent investigation reported no statistically significant differences in the dynamics of peripheral blood NK cells before and after stereotactic ablative body radiotherapy for lung or liver metastases in patients with oligo-metastatic cancers. Instead, a notable increase was observed in T-lymphocytes (CD3+CD19−), T-helper cells (CD3+CD4+), activated cytotoxic T-lymphocytes (CD3+CD8+HLA−DR+), and activated T-helpers (CD3+CD4+HLA−DR+) [64]. In a recent study, molecular subtyping using mRNA and miRNA networks was employed to investigate the molecular basis of resected colorectal liver oligometastases. Three distinct oligo-metastatic subtypes were identified: immune-enriched, stroma-enriched, and canonical. The immune-enriched subtype exhibited limited metastases with heightened cytotoxic immune responses, indicative of an indolent and immunologically balanced state. Conversely, the stroma-enriched subtype displayed pro-metastatic pathways linked to angiogenesis (*VEGFA* amplification) and unfavorable clinical outcomes. The molecular subtype categorized as canonical, linked to *E2F* (E2 transcription Factor)/*MYC* (Myelocytomatosis oncogene), DNA damage-related pathways, and cell cycle signaling, exhibited diverse patterns of metastatic recurrence and clinical outcomes [48]. In our view, substantial work remains in these aspects, necessitating improvements in study sample sizes, patient selection based on cancer types and comorbidity presence, and, when feasible, characterization of the tumor microenvironment for all available lesions in the same patient.

**Figure 2:**
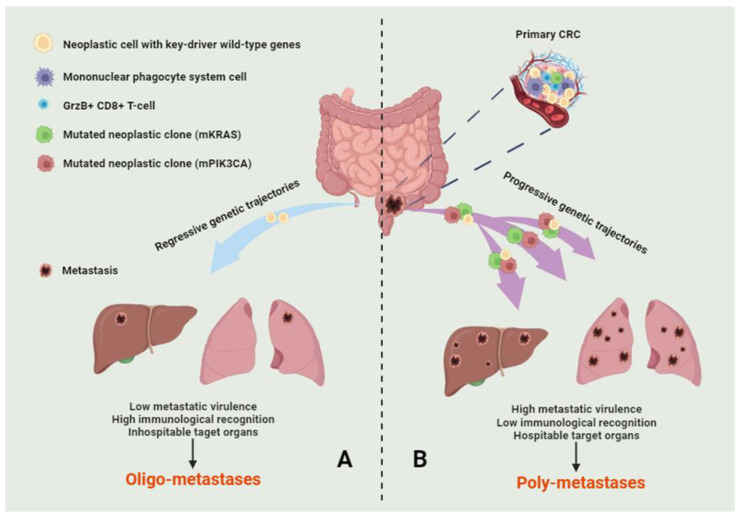
A multifactorial theory on the genesis of oligo-metastatic disease. The combination of genetic and immunological events may underlie oligo-metastatic (**A**) and poly-metastatic (**B**) disease in colorectal cancer. Specifically, regression of cellular clones with key driver gene mutations, a genetic background predisposing to lower metastatic virulence, and the host’s lymphocytes’ enhanced recognition of neoplastic cells may reduce the extent of the metastatic phenomenon. The figure illustrates the relevant cellular populations and genetic trajectories. In this context, only dynamic genetic assessments can shed light on the most significant events.

An image illustrating the putative phenomena underlying OMD is presented in Figure 2.

## 5. Conclusions and Future Perspectives

OMD challenges the traditional view of metastasis as an incurable stage of cancer. In this manuscript, we have explored the concept of OMD in oncology, emphasizing its less aggressive biological behavior and extended survival times. We have also discussed the pivotal role of genetics in characterizing OMD. OMD represents a unique subset of metastatic tumors, and its identification at presentation is crucial. This identification provides a therapeutic window for aggressive local interventions when feasible, potentially achieving long-term disease control or even a cure.

Genetic profiling and dynamic assessments are essential tools for understanding the genetic underpinnings of OMD. These approaches enable us to uncover specific genetic alterations associated with the less aggressive phenotype of oligo-metastatic lesions. Next-generation sequencing (NGS) and liquid biopsies provide valuable insights into genetic dynamics, allowing us to track the evolution of genetic alterations as the disease progresses.

Despite their promise, there are significant limitations to genetic assessments in OMD, including tumor heterogeneity, spatial and temporal variability, technical challenges in biopsy collection, interpretation of genetic data, and ethical considerations. Addressing these challenges requires ongoing research, innovation in technology, and collaboration among multidisciplinary teams.

## Data Availability

This article does not include original data or datasets.
